# Surveillance of Post-Vaccination Side Effects of COVID-19 Vaccines among Saudi Population: A Real-World Estimation of Safety Profile

**DOI:** 10.3390/vaccines10060924

**Published:** 2022-06-10

**Authors:** Abdulaziz Ibrahim Alzarea, Yusra Habib Khan, Ahmed D. Alatawi, Abdullah Salah Alanazi, Sami I. Alzarea, Muhammad Hammad Butt, Ziyad Saeed Almalki, Abdullah K. Alahmari, Tauqeer Hussain Mallhi

**Affiliations:** 1 Department of Clinical Pharmacy, College of Pharmacy, Jouf University, Sakaka 72388, Saudi Arabia; aizarea@ju.edu.sa (A.I.A.); adalatawi@ju.edu.sa (A.D.A.); asdalananzi@ju.edu.sa (A.S.A.); 2 Health Sciences Research Unit, Jouf University, Sakaka 72388, Saudi Arabia; 3 Department of Pharmacology, College of Pharmacy, Jouf University, Sakaka 72341, Saudi Arabia; samisz@ju.edu.sa; 4 Faculty of Pharmacy, University of Central Punjab, Lahore 54000, Pakistan; hmdbut@ucp.edu.pk; 5 Department of Clinical Pharmacy, College of Pharmacy, Prince Sattam Bin Abdulaziz University, Al-Kharj 11942, Saudi Arabia; z.almalki@psau.edu.sa (Z.S.A.); a.alahmari@psau.edu.sa (A.K.A.)

**Keywords:** COVID-19, vaccines, Oxford-AstraZeneca, Pfizer-BioNTech, safety profile, side effects, surveillance, pharmacovigilance, Saudi Arabia

## Abstract

Vaccines are considered to be the most beneficial means for combating the COVID-19 pandemic. Although vaccines against SARS-CoV-2 have demonstrated excellent safety profiles in clinical trials, real-world surveillance of post-vaccination side effects is an impetus. The study investigates the short-term side effects following the administration of the Pfizer-BioNTech and Oxford-AstraZeneca vaccines in Saudi Arabia. A cross-sectional quantitative study was conducted among the general population with age ≥ 18 years, from five regions (Central, Northern, Eastern, Southern, and Western Regions) of Saudi Arabia for a period of 6 months (July to December 2021). A self-administered study instrument was used to record the side effects among the COVID-19 vaccine recipients. Of the total 398 participants (males: 59%), 56.3% received Pfizer and 43.7% were vaccinated with AstraZeneca. Only 22.6% of respondents received the second dose of the COVID-19 vaccines. The most commonly reported side effects were pain at the injection site (85.2%), fatigue (61.8%), bone or joint pain (54.0%), and fever (42.5%). The average side effects score was 3.4 ± 2.2. Females, young people, and Oxford-AstraZeneca recipients had a higher proportion of side effects. The Oxford-AstraZeneca vaccine recipients complained more about fever (*p* < 0.001), bone and joint pain (*p* < 0.001), fatigue (*p* < 0.001), loss of appetite (*p* = 0.001), headache (*p* = 0.008), and drowsiness (*p* = 0.003). The Pfizer-BioNTech vaccinees had more pain and swelling at the injection site (*p* = 0.001), and sexual disturbance (*p* = 0.019). The study participants also reported some rare symptoms (<10%) including heaviness, sleep disturbance, fainting, blurred vision, palpitations, osteomalacia, and inability to concentrate. This study revealed that both Pfizer-BioNTech and Oxford-AstraZeneca administration was associated with mild to moderate, transient, short-lived side effects. These symptoms corroborate the results of phase 3 clinical trials of these vaccines. The results could be used to inform people about the likelihood of side effects based on their demographics and the type of vaccine administered. The study reported some rare symptoms that require further validation through more pharmacovigilance or qualitative studies.

## 1. Introduction

Since the onset of the COVID-19 pandemic in 2019, all countries have been constantly striving to curb the pace of disease transmission. In this context, various measures including movement restrictions, social isolation, and lockdown were undertaken across the world. Moreover, global researchers continue to leave no stone unturned to find specific therapies for the management of COVID-19 patients [1,2]. However, some countries have been hit by the second and third waves immediately after easing the lockdown or spatial distancing restrictions [3]. On the other hand, the emergence of novel variants of SARS-CoV-2 has contributed substantially to the rampant increase in cases [4]. However, various health authorities around the globe have urged and continue to urge their populations to vaccinate against COVID-19, and collectively they consider immunization among the best ways to fight the pandemic and curb disease mortality and morbidity [5].

The immunization effort is the largest vaccination campaign globally and ever. Immunization is the most effective and safest approach to establishing significant herd immunity. All the countries are diligently attempting to optimally vaccinate their population, with some resource-limited nations and communities struggling to do so. The mRNA-based and adenovirus-vectored vaccines against COVID-19 have demonstrated satisfactory efficacy and safety in clinical trials [6]. However, there is a dearth of investigations ascertaining the safety data of these vaccines in real-world settings. The continuous emergence of new variants has forced many countries to offer booster doses to their population. Some countries are also administering heterologous booster regimens based on the availability of the vaccines [7]. The mass coverage of the COVID-19 vaccine has shown variable safety profiles of vaccines [8]. Since the available evidence suggest substantial concerns of the general population about vaccine safety [9], the pharmacovigilance monitoring of vaccines remains essential to improve safety profiles and enhance public trust.

The Kingdom of Saudi Arabia (KSA) has approved four vaccines, including two nonreplicating viral vector-based vaccines (Oxford AstraZeneca, Janssen), and two RNA-based vaccines (Pfizer/BioNTech, Moderna). According to a recent estimate, about 70% of the country’s population is fully vaccinated, and 75% of residents have received at least one dose [10]. In randomized control trials, these vaccines reported local (injection site pain, redness, and swelling) and systematic (muscle or joint pain, headache, and fatigue,) side effects [11]. The proportion of these side effects varied across the studies from 50% to 90%. Since existing data on the side effects of COVID-19 vaccines have emerged from health authorities, there is little information on real-world and patient-reported side effects after receiving COVID-19 vaccine doses. Despite impressive vaccination coverage around the globe, COVID-19 vaccine hesitancy still prevails in the general population particularly due to safety concerns, which may interfere with vaccination campaigns that may take some years until the end of the pandemic is officially declared. In this context, surveillance of vaccine safety is of paramount importance to mitigate the fear of vaccinations among the masses. On the other hand, the real-world data (RWD) on pharmacovigilance of the COVID-19 vaccine in Saudi Arabia are primarily based on the reporting from healthcare professionals [12], adolescents [13], the general population [14,15,16], and recipients of one type of vaccine [17,18]. This study aimed to investigate the short-term side effects of the administration of the COVID-19 vaccine doses among the general population in Saudi Arabia.

## 2. Methodology

### 2.1. Ethics Statement

This study was approved by the Local Committee of Bioethics (LCBE) at Jouf University, KSA (Approval number: 5-08-43). Before filling out the questionnaire, informed consent was obtained from all the participants, and data were anonymized prior to analysis.

### 2.2. Study Design and Location

This cross-sectional quantitative study was conducted among the general population of the five regions of Saudi Arabia. The data were collected for a period of 6 months (July to December 2021) from five geographical regions of the KSA including were Central, Northern, Eastern, Southern, and Western Regions.

### 2.3. Study Population

**Figure 1:**
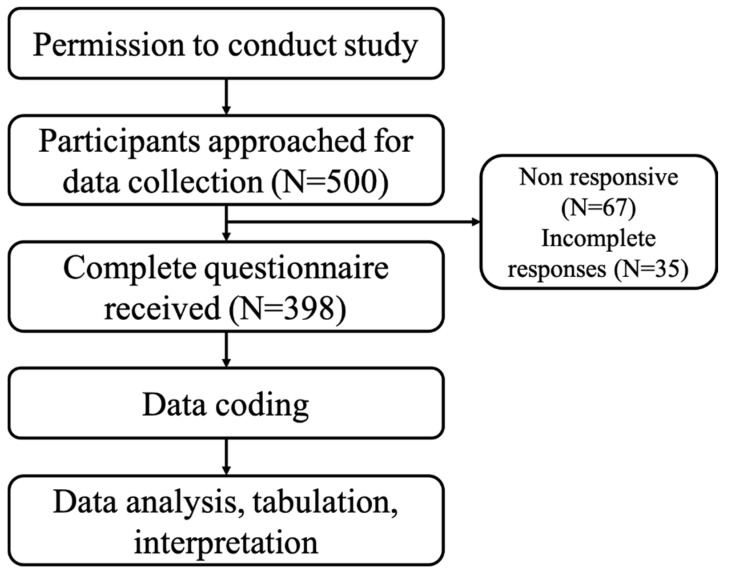
Study Flow Diagram.

All the Saudi residents with age ≥ 18 years, and those willing to participate were included in this survey. The participants who did not agree or those with ages less than 18 years were excluded from this study. The flow diagram for the current study is presented in Figure 1.

### 2.4. Validation and Reliability of Study Instrument

A 39-item questionnaire comprising three sections was constructed under the opinions of experts from health specialties (physicians, and hospital/community pharmacists). Following the face and content validity, the study instrument was administered in a small sample of 50 participants having equal distribution from all five regions (10 participants from each region). The internal consistency of the study tool was evaluated by alpha value, which was found at 0.845, indicating the suitability and reliability of the tool to evaluate the study objectives.

### 2.5. Components of Study Instrument

The COVID-19 vaccination in Saudi Arabia among the general population was initiated in early 2021 for both Oxford-AstraZeneca and Pfizer-BioNTech vaccines. A dual-language (Arabic and English) questionnaire was designed with the help of native Arabic speakers using forward–backward translation. The study questionnaire was divided into three sections. The first section collected the general information about the participants such as age, gender, geographic location, occupation, occupational field, average monthly income, marital status, nationality, and education. The second section inquired about the status of the COVID-19 vaccination among the participants (type and interval of vaccinations). The third section was comprised of the side effects profile. The side effects included in this section were pain, redness, and swelling at the injection site, fever, bone or joint pain, fatigue, loss of appetite, headache, nausea, drowsiness, sexual disturbance, shortness of breath, and others. The complete questionnaire was added in the Supplementary File (Supplementary File S2). All the participants were asked to respond against each side effect on a scale of “Yes”, “No”, and “Not Sure”. The option “Yes” was scored “1”, otherwise “zero”. The mean side effect score for each participant was calculated by the total number of side effects indicated by the respondent divided by the total number of side effects reported in this study (N = 27), resulting in a side effect score ranging from 0 to 27. The reported side effects were further stratified into four classes, namely, common (side effects indicated by >50% of the study population), moderately common (30–50%), uncommon (10% to <30%), and rare (<10%).

### 2.6. Data Collection

Using a convenient sampling technique, all the authors were asked to contact the general public in five regions of KSA. Informed consent was obtained from each participant before administering the survey. A brief overview of the study was given to the participants. All questionnaires were checked for completeness and transferred to the Microsoft spreadsheet for cleaning purposes.

### 2.7. Sample Size

The sample size was calculated on the basis of population size using the OpenEpi web-based sample size calculator. There were no statistical data available to calculate the number of participants, so we take the whole country’s population (around 30 million) as our population size [13]. The sample size of the 30 million population size comes out to be 385, with a 5% confidence limit, 50% anticipated % frequency, and design effect as 1 [19].

### 2.8. Statistical Analysis

Following cleaning and coding, all data were inserted into SPSS version 23. The data were descriptively presented as frequency along with percentage, and means with standard deviations. The categorical data (comparison of side effects across demographics and type of COVID-19 vaccine) were compared using Chi-square (χ2) test (if at least 80% of cells had expected frequencies of 5 or more than 5) or Fisher’s exact test (if <80% of cells had frequencies of 5 or more than 5) [20]. The continuous variables, such as mean score between two or more than two groups, were analyzed using independent student t-test and one-way ANOVA, respectively. A linear regression model was applied between type of vaccine and individual side effects to find the R^2^ values. The Coefficient of determination (CoD) was also estimated while comparing the frequencies of side effects across demographics and vaccine types. A significance level of *p* < 0.05 value was assessed in all analyses.

## 3. Results

### 3.1. Demographic Profile

Of the total 398 participants, 59% were males, 49.5% were from the central region of the KSA, and 57.8% were government sector employees. The majority of the participants were aged from 18 to 35 years (44.5%) and 36 to 50 years (44.5%). About two-thirds (60.6%) of participants were from non-medical occupations. The majority of the participants received Pfizer (56.3%) followed by AstraZeneca (43.7%). Only 22.6% had received a second dose of the COVID-19 vaccine at the time of the study. All demographic details are presented in Table 1.

### 3.2. Side Effects Profile

**Figure 2:**
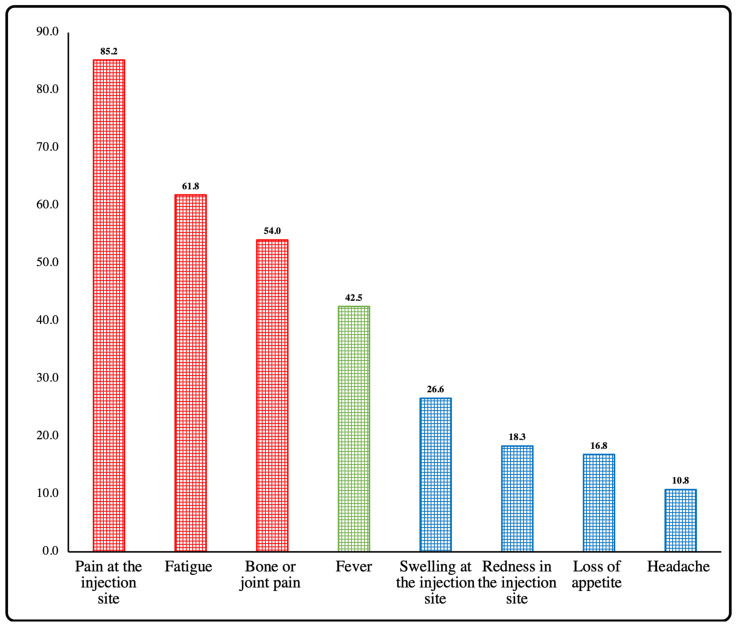
Percentage of common/moderately common, and uncommon side effects among study participants.

**Figure 3:**
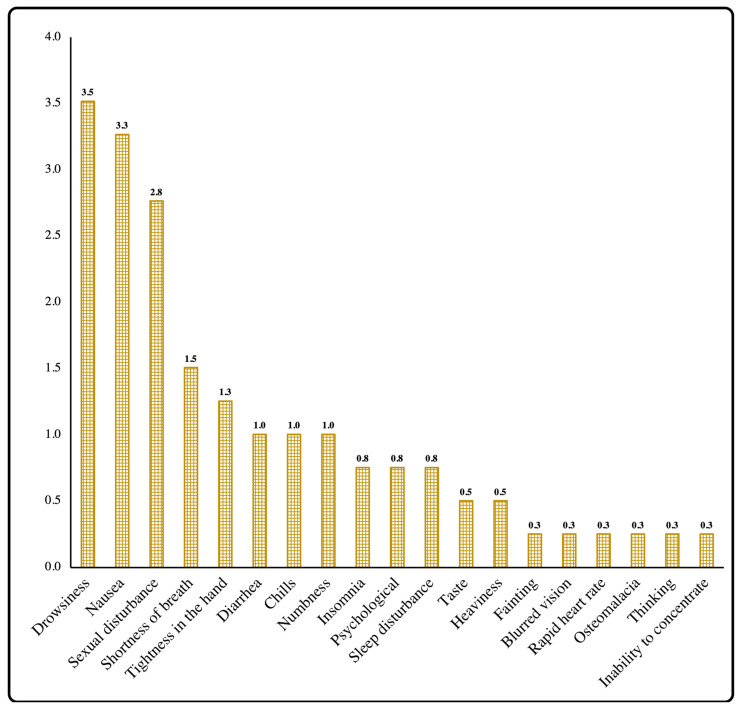
Percentage of rare side effects among study participants.

The common side effects (presented in >50% of the study participants) were pain at the injection site, fatigue, and bone or joint pain, while fever was a moderately common side effect (presented in 30–50% of the study participants) presented in 42.5% of the participants. The uncommon side effects (presented in 10–30% of the study participants) were swelling or redness at injection site, loss of appetite, and headache (Figure 2). However, drowsiness, nausea, sexual disturbances, shortness of breath, tightness in hands, diarrhea, etc., were rarely reported side effects in this study (Figure 3).

Only 4.5% (n = 18) of the vaccinees reported no side effects. The average side effect score among the study participants was 3.4 ± 2.2. Overall, 80 (20.15%) participants reported one side effect, which was the most frequent response, followed by responses of three (n = 68, 17.1%), two (n = 63, 15.8%), five (n = 54, 13.6%), four (49, 12.3%), six (28, 7%), seven (n = 22, 5.5%), eight (n = 7, 1.8%), nine (n = 6, 1.5%), and ten (n = 3, 0.8%) side effects. The females scored higher for side effect frequency (*p* < 0.001) than males (4.04 ± 2.33 versus 2.98 ± 2.0). The respondents with age 18–35 years, businessman, divorced, non-medical, non-Saudis, and monthly income of <5 thousand SAR were significantly associated with higher SE scores. The participants who received the Pfizer COVID-19 vaccine reported fewer side effects (2.74 ± 2.07) as compared to recipients of AstraZeneca (4.16 ± 2.08) (Table 2).

### 3.3. Association of Side Effects among Types of Vaccine

The pain at the injection site and sexual disturbances were significantly associated with the Pfizer vaccine, while fever, bone or joint pain, fatigue, loss of appetite, headache, and drowsiness were significantly associated with the AstraZeneca vaccine (Table 3).

### 3.4. Association of Individual Side Effects across Demographics

The male gender was significantly associated with fever, joint pain, injection site pain, fatigue, and sexual disturbances. However, females reported swelling at the injection site, redness at the injection site, loss of appetite, and headache. Swelling at the injection site, pain at the injection site, and sexual disturbance were more common among Pfizer vaccine recipients, while fever, fatigue, redness at the injection site, joint pain, loss of appetite, and headache were more common among AstraZeneca recipients (Table 4). 

## 4. Discussion

The COVID-19 pandemic is a life-threatening global health crisis that has resulted in the implementation of various preventive measures including lockdowns, contact tracing, quarantine, isolation, and social distancing. However, these measures were implemented to varying degrees across the globe and provided disparate effectiveness in controlling disease transmission due to various challenges [21,22,23]. These findings urged the global health authorities to divert their focus to discovering and using effective and safe vaccines [24].

The World Healthcare Organization (WHO) approved the first vaccine (Pfizer/BioNTech) through emergency validation on 31 December 2020. Currently, around 200 COVID-19 vaccines are at various stages of pre-clinical and clinical trials [25]. Since vaccines were produced in a record time, various researchers have prioritized the investigations of their safety profile. Although the safety profiles of vaccines are satisfactory in clinical trials, real-world data are of paramount importance, as trials are accompanied by several limitations. An important limitation is the lack of participation of the vulnerable population in the clinical trials, i.e., elderly people, who were targeted as a priority group for the COVID-19 vaccination. Furthermore, the widespread rumors and misconceptions about the safety of these vaccines have created some sort of fear among the general population [26]. Since the real-world studies differ from RCTs in terms of various factors, active post-marketing pharmacovigilance of the COVID-19 vaccines turns out to be a major concern of the international community.

According to a recent estimate, Saudi Arabia recorded 752,078 confirmed cases of COVID-19 with 9060 deaths and 63,202,128 vaccine doses until 13 April 2022 [27]. The COVID-19 vaccination campaign in Saudi Arabia was rolled out in December 2020 immediately after the emergency authorization of the first vaccine (Pfizer-BioNTech) by the United States Food and Drug Administration (FDA). Since the inception of the vaccination campaign, no serious adverse effects were reported following the administration of the COVID-19 doses. However, the occurrence of blood clots or thrombosis along with low platelet count was reported by the Saudi-FDA among Oxford-AstraZeneca recipients [12].

Our surveillance report did not indicate any serious adverse events among the general population who has been vaccinated with either single or two doses of the COVID-19 vaccines. The commonly reported side effects in this study were pain at the injection site, fatigue, and bone or joint pain. These findings are consistent with another study conducted in Saudi Arabia reporting injection site pain in 80.6% of vaccine recipients. However, the same study reported lower incidences of fatigue (20.1%) and bone or joint pain (1%) after the second dose of the vaccine [18]. Another study conducted in Nigeria reported similar findings as pain at injection site, fatigue, and fever as the most common side effects [28]. A review study by Rahman, et al. reported injection site pain, chills, joint pain, fatigue, headache, and muscle pain [29]. A study reporting side effects of the COVID-19 vaccine from 22 Arab countries reported tiredness (59%), injection site pain (58%), laziness (46%), headache (45%), myalgia (41%), fever (39%), joint pain (38%), chills (28%), dizziness (28%), anxiety and sleep disorders (27%), and numbness (21%) as the most common side effects [30].

The difference in the incidence might be attributed to the fact that authors have reported tiredness and whole-body pain as separate symptoms, which were present in 15.3% and 6.5% of respondents, respectively. There is a high propensity that respondents might confuse the understanding of fatigue with these two symptoms. It is pertinent to mention that fatigue and joint pain were associated with AstraZeneca administration in our study, while all participants in other studies received the doses of the Pfizer vaccine [18]. Alamer et al. also conducted a study to investigate the side effects among 12- to 18-year-old persons receiving the Pfizer vaccine in Saudi Arabia and reported fatigue in 67% of participants [13]. However, joint and bone pain were reported in only 2% of participants in their study. Alhazmi et al. conducted an online questionnaire-based study in Saudi Arabia and reported pain and redness at the injection site (85%), fatigue (90%), fever (66%), and headache (62%) as common side effects after COVID-19 doses. Though these results corroborate the findings of our study, the incidence of joint and bone pain was lower (2%) than what we reported in this study [15]. Another study on the safety and reactogenicity of the COVID-19 vaccines in Saudi Arabia reported joint and muscle pain in 30.5% of Oxford-AstraZeneca vaccinees within seven days of the first dose [16]. The side effects reported and their frequencies in our study are also in line with other studies conducted around the world [31]. Since the adverse effects of the COVID-19 vaccines are reported to be a reason for vaccine hesitancy, it is important to note that the participants in the placebo arms of vaccine trials also reported a substantial number of side effects, referring to the nocebo effect, which accounted for 76% of systematic side effects after the first dose and 51.8% after the second dose [32]. These high nocebo responses should be considered in public vaccination programs.

The fatigue and fever symptoms were found to be associated with Oxford-AstraZeneca administration and these results are aligned with other studies [12,14,15,17]. Our analysis revealed a high proportion of side effects among AstraZeneca recipients, and these findings are consistent with previous investigations [12,14]. The Oxford-AstraZeneca was associated with fever, bone and joint pain, fatigue, loss of appetite, headache, and drowsiness. On the other hand, the Pfizer vaccine was associated with pain at the injection site and sexual disturbances. Injection site pain is reported in up to 90% of Pfizer vaccinees in Saudi Arabia [13] and is considered the most common side effect of this vaccine. It is pertinent to mention that sexual disturbances were reported by 11 males in our study. However, the nature and severity of this side effect were not explained by the respondents. It is important to note that none of the studies reported the association of the COVID-19 vaccine with male and female reproductive health. There have been concerns among the public about the impact of vaccines on sperm and infertility, mainly driven by vocal conspiracy theorists [33]. Lifshitz et al. investigated the impact of the Pfizer vaccine on the semen analysis parameters and found these parameters within the normal limit among vaccinated men, strengthening the notion that Pfizer’s vaccine is safe among males [34]. These findings necessitate the need to educate the community regarding misleading narratives of the COVID-19 vaccines on reproductive health.

The common side effects reported in our study are not bothersome as they are the indications that the vaccines are efficiently working to provoke reactions in the immune system. Our analysis showed that AstraZeneca recipients had more side effects than Pfizer vaccinees. However, there is no evidence per se to support that side effects are more common with AstraZeneca vaccines, as a recent meta-analysis indicated a higher incidence of side effects among Sputnik recipients [31]. The frequency and severity of side effects after the COVID-19 vaccines are primarily linked with the recipient‘s demographics. Our study showed a high frequency of the side effects among females as compared to males. These findings are in contrast with the results of another study conducted in Saudi Arabia where males presented with more symptoms than females after receiving their vaccines [16]. However, our results are in agreement with another study conducted on the Saudi population where females were 3.72 times more likely to have major side effects than males [12]. The higher incidence of post-vaccination side effects among females has been well discussed on the basis of biological mechanisms. The development of higher antibody responses and mounting of stronger and more rapid innate and adaptive immune responses among females explain the high frequency of adverse reactions among them [35]. It is interesting to note that businessmen reported more side effects in our analysis. Since the business community is markedly affected by the pandemic-associated controlling measures, the higher level of anxiety and worry among this group may be related to increased reporting of vaccine-associated side effects [36]. Likewise, the higher frequency of side effects among divorcees may also be linked to their psychological health during the pandemic. Unlike other investigations [17,31], our analysis did not show any association of occupational field (health and non-health professions) with the incidence of side effects. This might be associated with less participation of healthcare professionals (n = 157) in our study. The frequency and severity of the side effects after the COVID-19 vaccines are associated with recipients’ characteristics such as gender, age, previous exposure to COVID-19 infection, and immunocompetency [13]. These characteristics must be considered while informing the general population about the safety profile of the vaccines.

The results of this study may contribute to enhance the public confidence in the safety profile of the COVID-19 vaccines, which may result in the acceleration of the vaccine coverage process. However, the findings of this study must be interpreted in light of a few limitations. Convenience sampling may limit the generalizability of the findings. Since the results of this study are based on self-reporting, information bias such as reporting and recall bias cannot be disregarded in this study. Moreover, the reported symptoms were not verified and there is a propensity to incorrectly blame the side effects on the vaccines. There was less participation from the eastern and southern regions of Saudi Arabia, which hinders the generalizability of the findings throughout the country. This study did not report data on the onset, duration, and severity of the side effects. Moreover, the long-term effect of the vaccines was not investigated in this analysis. However, our study provides a list of rare side effects that must be assessed in future studies. Although we achieved the sample size in this study, a large sample may produce variable findings and will increase the statistical power of this study. The replication of the analyses in a larger population is warranted. Last but not least, those who experienced side effects might have been more interested in participating than those who did not; therefore, the overall prevalence of side effects in this study might be over-estimated. The random sampling across the country may produce variable findings and can shift frequent side effects to less frequent ones. Nevertheless, this study confirms the safety of the COVID-19 vaccines, and its findings can be utilized in creating awareness among individuals with vaccine hesitancy and ambivalence.

## 5. Conclusions

This study revealed that the COVID-19 vaccines are associated with mild to moderate, transient, and short-lived side effects that are aligned with the safety reports of phase 3 clinical trials of these vaccines. Pain at the injection site, fatigue, bone or joint pain, and pyrexia were commonly reported side effects. The female gender, young people, and Oxford-AstraZeneca recipients had a significantly higher proportion of side effects. The Oxford-AstraZeneca vaccinees complained more of fever, bone and joint pain, fatigue, loss of appetite, headache, and drowsiness. The Pfizer-BioNTech vaccine recipients had a significantly higher frequency of injection site pain. The data from this study could be used as part of a larger and growing body of evidence regarding the likelihood of side effects based on their demographics and the type of vaccine administered. It is important to note that various rare symptoms are reported in this study that require further validation through more pharmacovigilance or qualitative studies. A larger study with random sampling would likely detect the rare and uncommon symptoms observed in this study but would likely do so more frequently, thereby allowing for comparisons among subpopulations. Furthermore, some rare symptoms may be so infrequent that they are too rare to have been detected by this study (n = 398), such as rare side effect symptoms that affect one or fewer persons per 400 (e.g., <2.5 participants per 1000 persons).

## Figures and Tables

**Table 1:** Demographic data of study participants (N = 398).

Variables	Frequency (N)	Percentage (%)
***Age***		
18–35 years	177	44.5%
36–50 years	177	44.5%
>50 years	44	11.0%
***Gender***		
Male	235	59.0%
Female	163	41.0%
***Geographic location***		
Central Region	197	49.5%
Northern Region	122	30.7%
Eastern Region	21	5.3%
Southern Region	6	1.5%
Western Region	52	13.1%
***Occupation***		
Students	46	11.6%
Private sector employees	51	12.8%
Government sector employees	230	57.8%
Retired	27	6.8%
Own Business	44	11.0%
***Occupational field***		
Medical	157	39.4%
Non-medical	241	60.6%
***Average monthly income***		
<5 thousand SAR	108	27.1%
5–15 thousand SAR	141	35.4%
15–20 thousand SAR	71	17.8%
>20 thousand SAR	78	19.6%
***Marital status***		
Married	276	69.3%
Single	107	26.9%
Divorced	15	3.8%
***Nationality***		
Saudi	382	96.0%
Non-Saudi	16	4.0%
***Highest Certificate Obtained***		
High school	48	12.1%
Bachelor’s degree	222	55.8%
Masters	38	9.5%
Ph.D.	60	15.1%
Post-secondary diploma	30	7.5%
***Type of vaccine receive***		
Pfizer	224	56.3%
AstraZeneca	174	43.7%
***Second dose received***		
Yes	90	22.6%
No	308	77.4%
***Time recommended for 2nd dose***		
3 weeks	224	56.3%
3 months	174	43.7%

**Table 2:** Mean side effect score among demographics (N = 398).

Variables	N	Mean ± S.D.	*p*-Value
***Age***			0.181
18–35 years	177	3.59 ± 2.13	
36–50 years	177	3.19 ± 2.21	
51–65 years	44	3.14 ± 2.28	
***Gender***			**<0.001**
Male	235	2.89 ± 1.95	
Female	163	4.04 ± 2.33	
***Geographic location***			0.390
Central	197	3.15 ± 2.24,	
Northern	122	3.57 ± 2.26	
Eastern	21	3.33 ± 1.77	
Southern	6	3.83 ± 1.6	
Western	52	3.63 ± 2	
***Occupation***			**<0.001**
Student	46	3.35 ± 1.98	
Private sector employee	51	3.92 ± 2.28	
Government sector employee	230	3.2 ± 2.08	
Retired	27	2.19 ± 1.88	
Own Business	44	4.32 ± 2.57	
***Occupational field***			0.205
Medical	157	3.22 ± 2.06	
Non-medical	241	3.46 ± 2.27	
***Average monthly income***			0.062
<5 thousand SAR	108	3.7 ± 2.31	
5–15 thousand SAR	141	3.45 ± 2.09	
15–20 thousand SAR	71	2.85 ± 1.93,	
>20 thousand SAR	78	3.19 ± 2.35,	
***Marital Status***			**0.006**
Married	276	3.17 ± 2.12	
Single	107	3.67 ± 2.21	
Divorced	15	4.73 ± 2.71	
***Nationality***			**0.009**
Saudi	382	3.3 ± 2.18	
Non-Saudi	16	4.75 ± 1.95	
***Highest Certificate Obtained***			0.718
High school	48	3.35 ± 2.06	
Bachelor’s degree	222	3.37 ± 2.23	
Masters	38	2.92 ± 2.12	
Ph.D.	60	3.57 ± 2.36	
Post-secondary diploma	30	3.43 ± 1.85	
***Type of vaccine receive***			**<0.001**
Pfizer	224	2.74 ± 2.07	
AstraZeneca	174	4.16 ± 2.08	

Bold represents *p*-value was significant at < 0.05. ***Independent student t-test*** was used to compare the mean score between the two groups. ***One-way ANOVA*** was used to compare the mean score between more than two groups. Post hoc analysis (Tukey’s HSD): ***Occupation,*** *Private sector employee* vs. *Retired (p = 0.006); Government sector employee* vs. *Own Business (p = 0.013); Own Business* vs. *Retired (p = 0.001)* ***Average monthly income****, <5 thousand SAR* vs. *15–20 thousand SAR (p = 0.05). **Marital Status**, Married* vs. *Divorced (p = 0.018).*

**Table 3:** Association of the side effects with the type of Vaccine.

Side Effect	Total (398)	Pfizer (N = 224)	AstraZeneca (N = 174)	*p*-Value	R^2^
	N (%)	N (%)	N (%)		
Pain at the injection site	339 (85.2%)	203 (90.6%)	136 (78.20%)	**0.001**	0.030
Redness at the injection site	73(18.3%)	34 (15.2%)	39 (22.40%)	0.064	0.009
Swelling at the injection site	106 (26.7%)	64 (28.6%)	42 (24.10%)	0.321	0.002
Fever	169 (42.5%)	51 (22.8%)	118 (67.80%)	**<0.001**	0.204
Bone or joint pain	215 (38.4%)	86 (38.4%)	129 (74.10%)	**<0.001**	0.127
Fatigue	246 (54.0%)	100 (44.6%)	146 (83.90%)	**<0.001**	0.161
Loss of appetite	67 (16.8%)	25 (11.2%)	42 (24.10%)	**0.001**	0.030
Headache	43 (10.8%)	16 (7.1%)	27 (15.50%)	**0.008**	0.018
Sexual disturbance	11 (2.8%)	10 (4.5%)	1 (0.60%)	**0.019**	0.014
Drowsiness	14 (3.5%)	4 (1.8%)	10 (5.70%)	**0.033**	0.011
Nausea	13 (3.3%)	7 (3.1%)	6 (3.40%)	0.857	0.000
Shortness of breath	6 (1.5%)	3 (1.3%)	3 (1.70%)	0.775	0.000
Diarrhea	4 (1.0%)	2 (0.9%)	2 (1.10%)	1.000	0.000
Chills	4 (1.0%)	2 (0.9%)	2 (1.10%)	1.000	0.000
Insomnia	3 (0.8%)	2 (0.9%)	1 (0.60%)	1.000	0.000
Tightness in the hand	5 (1.3%)	1 (0.4%)	4 (2.30%)	0.173	0.007
Numbness	4 (1.0%)	2 (0.9%)	2 (1.10%)	1.000	0.000
Psychological	3 (0.8%)	1 (0.4%)	2 (1.10%)	0.583	0.002
Taste	2 (0.5%)	0 (0.0%)	2 (1.10%)	0.191	0.007
Heaviness	2 (0.5%)	0 (0.0%)	2 (1.10%)	0.191	0.007
Sleep disturbance	3 (0.8%)	0 (0.0%)	3 (1.70%)	0.083	0.010
Fainting	1 (0.3%)	0 (0.0%)	1 (0.60%)	0.437	0.003
Blurred vision	1 (0.3%)	0 (0.0%)	1 (0.60%)	0.437	0.003
Rapid heart rate	1 (0.3%)	1 (0.4%)	0 (0.00%)	1.000	0.002
Osteomalacia	1 (0.3%)	0 (0.0%)	1 (0.60%)	0.437	0.003
Thinking	1 (0.3%)	0 (0.0%)	1 (0.60%)	0.437	0.003
Inability to concentrate	1 (0.3%)	0 (0.0%)	1 (0.60%)	0.437	0.003

Bold represents *p*-value was significant at < 0.05. Chi-square test or Fisher’s Exact Test were used to check the association of side effects with type of vaccine. Linear regression was applied to find R^2^ values.

**Table 4:** Association of individual side effects among demographics.

Variables	Pain	Fatigue	Joint Pain	Fever	Redness	Swelling	Loss of Appetite	Headache	Drowsiness	Nausea	Sexual Disturbance	Shortness of Breath
	N (%)	N (%)	N (%)	N (%)	N (%)	N (%)	N (%)	N (%)	N (%)	N (%)	N (%)	N (%)
**Gender**												
Male	191 (56.3%)	132 (53.7%)	114 (53%)	87 (51.5%)	31 (42.5%)	49 (46.2%)	19 (28.4%)	16 (37.2%)	6 (42.9%)	6 (46.1%)	11 (100%)	1 (16.6%)
Female	148 (43.7%)	114 (46.3%)	101 (47%)	82 (48.5%)	42 (57.5%)	57 (53.8%)	48 (71.6%)	27 (62.8%)	8 (57.1%)	7 (53.9%)	0 (0%)	5 (83.4%)
*p-value*	0.009 *	0.005 *	0.008 *	0.008 *	0.001 *	0.002 *	<0.001 *	0.002 *	0.21	0.337	0.005 *	0.033 *
**Geographic location**												
Central	179 (52.8%)	103 (41.9%)	94 (43.7%)	65 (38.5%)	35 (47.9%)	46 (43.4%)	36 (53.7%)	19 (44.2%)	6 (42.9%)	7 (53.9%)	9 (81.8%)	5 (83.3%)
Northern	91 (26.8%)	84 (34.1%)	72 (33.5%)	65 (38.5%)	29 (39.7%)	40 (37.7%)	22 (32.8%)	11 (25.6%)	4 (28.6%)	4 (30.8%)	2 (18.2%)	0 (0%)
Eastern	18 (5.3%)	17 (6.9%)	12 (5.6%)	10 (5.9%)	2 (2.7%)	4 (3.8%)	1 (1.5%)	2 (4.7%)	2 (14.3%)	0 (0%)	0 (0%)	0 (0%)
Southern	5 (1.5%)	5 (2%)	6 (2.8%)	4 (2.4%)	1 (1.4%)	2 (1.9%)	0 (0%)	0 (0%)	0 (0%)	0 (0%)	0 (0%)	0 (0%)
Western	46 (13.6%)	37 (15%)	31 (14.4%)	25 (14.8%)	6 (8.2%)	14 (13.2%)	8 (11.9%)	11 (25.6%)	2 (14.3%)	2 (15.4%)	0 (0%)	1 (16.7%)
*p-value*	0.003 *	0.002 *	0.037 *	0.004 *	0.272	0.378	0.42	0.125	0.624	0.907	0.267	0.441
**Age**												
18–35 years	155 (45.7%)	124 (50.4%)	104 (48.4%)	87 (51.5%)	36 (49.3%)	46 (43.4%)	35 (52.2%)	17 (39.5%)	7 (50%)	4 (30.8%)	3 (27.3%)	2 (33.3%)
36–50 years	150 (44.2%)	100 (40.7%)	89 (41.4%)	65 (38.5%)	28 (38.4%)	44 (41.5%)	28 (41.8%)	19 (44.2%)	6 (42.9%)	8 (61.6%)	8 (72.7%)	4 (66.7%)
51–65 years	34 (10%)	22 (8.9%)	22 (10.2%)	17 (10.1%)	10 (13.7%)	16 (11.1%)	4 (6%)	7 (16.3%)	1 (7.1%)	1 (7.7%)	1 (9.1%)	0 (0%)
*p-value*	<0.001 *	<0.001 *	0.038 *	0.008 *	0.622	0.151	0.298	0.259	0.928	0.627	0.258	0.678
**Occupation**												
Student	39 (11.5%)	30 (12.2%)	25 (11.6%)	22 (13%)	5 (6.8%)	9 (8.5%)	11 (16.4%)	6 (14%)	2 (14.3%)	0 (0%)	0 (0%)	1 (16.7%)
Private sector employees	43 (12.7%)	38 (15.4%)	37 (17.2%)	25 (14.8%)	11 (15.1%)	15 (14.2%)	10 (14.9%)	7 (16.3%)	2 (14.3%)	4 (30.8%)	0 (0%)	1 (16.7%)
Government sector employees	199 (58.7%)	135 (54.9%)	114 (53%)	92 (54.4%)	39 (53.4%)	59 (55.7%)	31 (46.3%)	22 (51.2%)	6 (42.9%)	7 (53.9%)	11 (100%)	4 (66.7%)
Retired	17 (5%)	9 (3.7%)	12 (5.6%)	5 (3%)	2 (2.7%)	6 (5.7%)	1 (1.5%)	3 (7%)	1 (7.1%)	0 (0%)	0 (0%)	0 (0%)
Own Business	41 (12.1%)	34 (13.8%)	27 (12.6%)	25 (14.8%)	16 (21.9%)	17 (16%)	14 (20.9%)	5 (11.6%)	3 (21.4%)	2 (15.4%)	0 (0%)	0 (0%)
*p-value*	0.001 *	0.001 *	0.028 *	0.017 *	0.007 *	0.287	0.007 *	0.898	0.719	0.191	0.082	0.848
**Profession**												
Medical	141 (41.6%)	94 (38.2%)	79 (36.7%)	57 (33.7%)	23 (31.5%)	39 (36.8%)	23 (34.3%)	16 (37.2%)	4 (28.6%)	7 (53.9%)	3 (27.3%)	2 (33.3%)
non-medical	198 (58.4%)	152 (61.8%)	136 (63.3%)	112 (66.3%)	50 (68.5%)	67 (63.2%)	44 (65.7%)	27 (62.8%)	10 (71.4%)	6 (46.2%)	8 (72.7%)	4 (66.7%)
*p-value*	0.036 *	0.521	0.232	0.045 *	0.125	0.514	0.347	0.751	0.397	0.28	0.402	0.758
**Monthly income**												
<5000 SAR	93 (27.4%)	75 (30.5%)	65 (30.2%)	51 (30.2%)	25 (34.2%)	29 (27.4%)	28 (41.8%)	12 (27.9%)	5 (35.7%)	4 (30.8%)	0 (0%)	4 (66.7%)
5000–15,000 SAR	120 (35.4%)	88 (35.8%)	82 (38.1%)	69 (40.8%)	24 (32.9%)	35 (33%)	25 (37.3%)	19 (44.2%)	6 (42.9%)	2 (15.4%)	3 (27.3%)	1 (16.7%)
15,000–20,000 SAR	57 (16.8%)	41 (16.7%)	36 (16.7%)	25 (14.8%)	10 (13.7%)	15 (14.2%)	6 (9%)	6 (14%)	1 (7.1%)	3 (23.1%)	0 (0%)	0 (0%)
>20,000 SAR	69 (20.4%)	42 (17.1%)	32 (14.9%)	24 (14.2%)	14 (19.2%)	27 (25.5%)	8 (11.9%)	6 (14%)	2 (14.3%)	4 (30.8%)	8 (72.7%)	1 (16.7%)
*p-value*	0.554	0.152	0.041 *	0.025 *	0.441	0.274	0.006 *	0.523	0.624	0.449	<0.001 *	0.156
**Marital status**												
Married	233 (68.7%)	159 (64.6%)	142 (66%)	105 (62.1%)	45 (61.6%)	72 (67.9%)	36 (53.7%)	27 (62.8%)	9 (64.3%)	9 (69.2%)	11 (100%)	4 (66.7%)
Single	95 (28%)	74 (30.1%)	62 (28.8%)	53 (31.4%)	24 (32.9%)	29 (27.4%)	26 (38.8%)	11 (25.6%)	5 (35.7%)	3 (23.1%)	0 (0%)	2 (33.3%)
Divorced	11 (3.2%)	13 (5.3%)	11 (5.1%)	11 (6.5%)	4 (5.5%)	5 (4.7%)	5 (7.5%)	5 (11.6%)	0 (0%)	1 (7.7%)	0 (0%)	0 (0%)
*p-value*	0.241	0.035 *	0.23	0.012 *	0.29	0.13	0.008 *	0.012 *	0.796	0.814	0.172	0.954
**Nationality**												
Saudi	326 (96.2%)	231 (93.9%)	201 (93.5%)	156 (92.3%)	70 (95.9%)	102 (96.2%)	60 (89.6%)	40 (93%)	12 (85.71%)	13 (100%)	11 (100%)	5 (83.3%)
non-Saudi	13 (3.8%)	15 (6.1%)	14 (6.5%)	13 (7.7%)	3 (4.1%)	4 (3.8%)	7 (10.4%)	3 (7%)	2 (14.29%)	0 (0%)	0 (0%)	1 (16.7%)
*p-value*	0.652	0.007 *	0.006 *	0.001 *	0.966	0.88	0.003 *	0.296	0.047 *	0.453	0.491	0.112
**Education**												
High school	39 (11.5%)	34 (13.8%)	28 (13%)	27 (16%)	6 (8.2%)	7 (6.6%)	11 (16.4%)	5 (11.6%)	2 (14.3%)	0 (0%)	0 (0%)	0 (0%)
Bachelor’s degree	191 (56.3%)	132 (53.7%)	118 (54.9%)	90 (53.3%)	43 (58.9%)	65 (61.3%)	42 (62.7%)	24 (55.8%)	7 (50.0%)	7 (53.8%)	1 (9.1%)	5 (83.3%)
Masters	28 (8.3%)	22 (8.9%)	19 (8.8%)	13 (7.7%)	7 (9.6%)	8 (7.5%)	3 (4.5%)	5 (11.6%)	1 (7.14%)	2 (15.4%)	0 (0%)	0 (0%)
Ph.D.	53 (15.6%)	42 (17.1%)	30 (14%)	27 (16%)	14 (19.2%)	16 (15.1%)	8 (11.9%)	5 (11.6%)	2 (14.29%)	3 (23.1%)	7 (63.6%)	1 (16.7%)
Post-secondary diploma	28 (8.3%)	16 (6.5%)	20 (9.3%)	12 (7.1%)	3 (4.1%)	10 (9.4%)	3 (4.5%)	4 (9.3%)	2 (14.29%)	1 (7.7%)	3 (27.3%)	0 (0%)
*p-value*	0.321	0.395	0.554	0.236	0.487	0.291	0.339	0.961	0.489	0.719	<0.001 *	0.727
**Vaccine**												
Pfizer	203 (59.9%)	100 (40.7%)	86 (40%)	51 (30.2%)	34 (46.6%)	64 (60.4%)	25 (37.3%)	16 (37.2%)	4 (28.57%)	7 (53.9%)	10 (90.9%)	3 (50%)
AstraZeneca	136 (40.1%)	146 (59.3%)	129 (60%)	118 (69.8%)	39 (53.4%)	42 (39.6%)	42 (62.7%)	27 (62.8%)	10 (71.43%)	6 (46.2%)	1 (9.1%)	3 (50%)
*p-value*	0.001 *	<0.001 *	<0.001 *	<0.001 *	0.064	0.321	0.001 *	0.008 *	0.033 *	0.857	0.019 *	0.755

* Statistically Significant. Chi-square test and Fisher’s Exact Test were used to check the association of side effects with demographics.

## Data Availability

Not applicable.

## Supplementary Materials

The following supporting information can be downloaded at: https://www.mdpi.com/article/10.3390/vaccines10060924/s1. R^2^ results were presented in Supplementary File S1, and Data collection form was added as Supplementary File S2.
