# Supplementary material for: Surveillance of Post-Vaccination Side Effects of COVID-19 Vaccines among Saudi Population: A Real-World Estimation of Safety Profile
Source: Vaccines (Basel). 2022 Jun 10;10(6):924. doi: 10.3390/vaccines10060924 (PMC9228257; doi:10.3390/vaccines10060924)
Supplement: Supplementary file 1 [file vaccines-10-00924-s001.zip › Supplementary file 1.pdf]

Supp Table S1; *Linear regression across demographics and individual side effects*

	Gender	Geographic location	Age	Occupation	Profession	Monthly income	Marital status	Nationality	Education	type of Vaccine
	R^2	R^2	R^2	R^2	R^2	R^2	R^2	R^2	R^2	R^2
Pain at the injection site	1.74	0.11	0.672	0.0144	1.1025	0.0016	0.0225	0.0529	0.3025	3.00
Redness at the injection site	2.56	0.31	0.026	1.2544	0.5929	0.2916	0.6084	0.0004	0.0064	0.90
Swelling at the injection site	2.46	0.07	0.212	0.6241	0.1089	0.1936	0.0841	0.0064	0.25	0.20
Fever	1.74	1.44	1.061	0.01	1.0201	1.8496	0.0256	2.5921	0.1681	20.40
Bone or joint pain	1.77	1.06	0.593	0.0784	0.36	1.9044	0.9216	1.8769	0.0169	12.70
Fatigue	1.93	2.40	2.372	0.0324	0.1024	1.2996	2.1025	1.8225	0.0289	16.10
Loss of appetite	7.90	0.24	0.757	0.0121	0.2209	2.6569	2.3104	2.1609	1	3.00
Headache	2.40	1.04	0.281	0.0529	0.0256	0.2704	1.2996	0.2704	0.0009	1.80
Sexual disturbance	1.96	1.02	0.152	0.0121	0.1764	3.3124	0.9025	0.1225	5.76	1.40
Drowsiness	0.40	0.05	0.073	0.0676	0.1764	0.2809	0.0049	1	0.0576	1.10
Nausea	0.23	0.01	0.084	0.0144	0.2916	0.1681	0.0484	0.1444	0.3249	0.00
Shortness of breath	1.14	0.08	0	0.2601	0.0225	0.5329	0.0081	0.64	0.0361	0.00
Diarrhea	0.49	0.55	0.017	0.0049	0.09	0.0361	0.3025	0.0441	0	0.00
Chills	0.11	0.13	0.017	0.0049	0.5329	0.81	0.3249	0.0441	0	0.00
Insomnia	0.02	1.21	0.194	0.3721	0.0121	0.09	0.2401	0.0324	0.1521	0.00
Tightness in the hand	0.19	0.12	0.053	0.8836	0.0001	0.0121	1.0609	0.0529	0.0961	0.70
Numbness	1.46	0.00	1.02	0.0049	0.09	0.9801	0.3025	0.0441	0.2025	0.00
Psychological	0.02	0.04	0.757	0.0484	1.1664	0.09	0.0036	1.69	0.8281	0.20

Taste	0.72	0.29	0.106	0.0025	0.0225	0.2809	2.8561	0.0225	0.1024	0.70
Heaviness	0.02	0.66	0.792	0.1521	0.0225	0.2209	1.3689	0.0225	0	0.70
Sleep disturbance	0.21	0.18	0	0.0036	1.1664	0.09	0.6241	0.0324	0.0169	1.00
Fainting	0.36	0.00	0.25	0.8649	0.3844	0.3721	0.2116	0.01	0.4624	0.30
Blurred vision	0.18	0.14	0.25	0.8649	0.1681	0.1089	0.2116	0.01	0.0484	0.30
Rapid heart rate	0.36	0.14	0.063	0.2025	0.1681	0.0196	0.0784	0.01	0.0529	0.20
Osteomalacia	0.18	1.28	0.063	0.2025	0.1681	0.0196	0.0784	0.01	0.4624	0.30
Thinking	0.18	0.14	0.063	0.0009	0.1681	0.64	0.0784	0.01	0.4489	0.30
Inability to concentrate	0.18	0.14	0.063	0.0009	0.1681	0.64	0.0784	0.01	0.4489	0.30

Linear regression was applied to find R^2 values.
