# Supplementary material for: Surveillance of Post-Vaccination Side Effects of COVID-19 Vaccines among Saudi Population: A Real-World Estimation of Safety Profile
Source: Vaccines (Basel). 2022 Jun 10;10(6):924. doi: 10.3390/vaccines10060924 (PMC9228257; doi:10.3390/vaccines10060924)
Supplement: Supplementary file 1 [file vaccines-10-00924-s001.zip › Supplementary file 2.pdf]

Surveillance of post-vaccination side effects of COVID-19 vaccines among Saudi Population: A real-world estimation of Safety Profile

This research project will help to understand the prevalence of side effects associated with the COVID-19 vaccine doses after first and second dose. It will just take 5 minutes from your precious time. Your response will greatly help us to find out which type of side effects were prevailing in the saudi population. Please fill this survey, if you consent to participate. Your cooperation will be greatly appreciated.

Section 1; Demographics

1. **Age**
   a) 18 - 35 years b) 36 - 50 years c) >50 years
2. **Gender**
   a) Male b) Female
3. **Geographic location**
   a) Central Region b) Northern Region c) Eastern Region d) Southern Region e) Western Region
4. **Occupation**
   a) Students b) Private sector employees c) Government sector employees d) Retired e) Own Business
5. **Occupational field**
   a) Medical b) non-medical
6. **Average monthly income**
   a) < 5 thousand SAR b) 5 - 15 thousand SAR c) 15 - 20 thousand SAR d) 20 thousand SAR
7. **Marital status**
   a) Married b) Single c) Divorced
8. **Nationality**
   a) Saudis b) Non-Saudis
9. **Highest Certificate Obtained**
   a) High school b) Bachelor's degree c) Masters d) Ph.D. e) Post-secondary diploma
10. **Type of vaccine receive**
    a) Pfizer b) AstraZeneca
11. **Second dose received**
    a) Yes b) No
12. **Time recommended for 2nd dose**
    a) 3 weeks b) 3 months

Section 2; Side effect Profile

Note; *Please tick the side effect which occurred after COVID-19 vaccination*

Side Effect	Yes	No	Side Effect	Yes	No
Pain at the injection site			Insomnia		
Redness at the injection site			Tightness in the hand		
Swelling at the injection site			Numbness		
Fever			Psychological		
Bone or joint pain			Taste		
Fatigue			Heaviness		
Loss of appetite			Sleep disturbance		
Headache			Fainting		
Sexual disturbance			Blurred vision		
Drowsiness			Rapid heart rate		
Nausea			Osteomalacia		
Shortness of breath			Thinking		
Diarrhea			Inability to concentrate		
Chills			Other		
